# Normal gains: estimators of learning rates in pretest-posttest settings

**DOI:** 10.3389/fpsyg.2026.1901493

**Published:** 2026-09-01

**Authors:** Jairo A. Navarrete-Ulloa, Valentina Giaconi, Gonzalo Contador

**Affiliations:** 1 Institute of Education Sciences, Universidad de O'Higgins, Rancagua, Chile; 2 Departamento de Matemática, Universidad Tecnica Federico Santa Maria, Valparaíso, Chile

**Keywords:** classical test theory, estimator bias, measurement error, nonlinear derived scores, normalized gains, pretest-posttest design, reliability

## Abstract

**Introduction:**

Nonlinear transformations of pretest and posttest scores are widely used in educational and psychological measurement to estimate group-level change, yet the statistical behavior of estimators derived from such transformations under measurement error remains poorly understood. We examine this problem in the context of normalized gains (ngains), a ratio-based transformation used to estimate group-level “learning rates” in pretest-posttest designs. Two standard estimation methods — the *average ngain of the group* ($\bar{ng}$) and the *ngain of the average learner* ($\hat{ng}$) — routinely produce different results. A prior study established a mathematical relationship between this discrepancy and the pretest-ngain correlation, interpreting it as a characterization of the learning process. The pretest-ngain correlation has itself sparked debate: researchers have argued it indicates that ngains favor high-pretest populations, undermining their validity as a measure of student growth.

**Methods:**

Using Classical Test Theory along with a rencently proposed statistical framework to analize ngains, we show that measurement error is one common cause behind both phenomena.

**Results:**

When measurement errors are absent, both $\bar{ng}$ and $\hat{ng}$ are unbiased and any discrepancy between them reflects only sampling variation. When measurement errors are present, $\bar{ng}$ acquires a systematic negative bias — consistently underestimating the true learning rate — while $\hat{ng}$ remains asymptotically unbiased. We further prove that measurement errors induce a spurious negative correlation between pretest scores and ngains, even when prior knowledge and learning capacity are statistically independent.

**Discussion:**

Such correlations may reflect insufficient instrument reliability rather than any inherent flaw in the transformation. These findings generalize beyond ngains: any nonlinear derived score computed from fallible instruments is susceptible to the same bias structure, and the analytical approach developed here offers a methodological template applicable to other ratio-based metrics in educational and psychological measurement. For applied researchers, we recommend computing both estimators and treating a large discrepancy as a warning sign, reporting instrument reliability alongside ngain estimates, and interpreting pretest-ngain correlations conditionally on reliability.

## 1 Introduction

Nonlinear transformations of observed scores are pervasive in educational and psychological measurement. Researchers routinely compute ratio-based, proportion-correct, or residualized scores from fallible pretest and posttest instruments, and use these derived quantities to estimate population-level change. Yet, a fundamental statistical question is seldom asked: how does measurement error propagate through nonlinear score transformations, and what are the consequences for the estimators derived from them? The present study addresses this question in a setting where the problem is both theoretically tractable and practically consequential — the estimation of group-level normalized gains (ngains) in pretest-posttest designs.

Normalized gains (ngains) have become a standard tool for measuring student growth in physics and mathematics education, yet a basic question remains unresolved: two commonly used methods for computing group-level ngains routinely produce different results, and the reasons for this discrepancy are poorly understood. The first method averages the individual ngain scores across students ($\bar{ng}$), while the second computes the ngain of the group's average learner ($\hat{ng}$). Prior studies have documented that these two methods often yield different empirical estimates (Bao, 2006; Marx and Cummings, 2007), but no study has systematically examined why. The present study argues that the answer lies in an overlooked source of variability: measurement errors in pretest and posttest scores.

Pretest-posttest designs are particularly valuable in educational research, as they enable researchers to assess the impact of teaching methodologies, educational tools, and individual variables—such as cognitive abilities, working memory, and math anxiety—on learning outcomes (Murnane and Willett, 2011). Estimating both individual and group-level gains is essential, as demonstrated by the extensive literature on value-added models (Leckie, 2018). The literature has developed various approaches to this end, ranging from traditional methods such as raw gain scores (Williams and Zimmerman, 1996; Trafimow, 2015), residualized gains (Bezruczko et al., 2016) and final knowledge status (Castellano and McCaffrey, 2019) to more sophisticated tools such as growth curve models (Liu and Perera, 2024; Commins et al., 2024). However, knowledge gains due to instruction are inherently challenging to estimate (Willett, 1988; Bonate, 2000), and a thorough understanding of the differences between estimation methods is crucial. The consequences of ignoring measurement error in pretest scores have been documented in related designs: Miyazaki et al. (2022) showed analytically that measurement error in the pretest biases treatment effect estimates in ANCOVA, with the direction of bias depending on reliability and the pretest group mean difference. The present study extends this line of inquiry to the nonlinear ngain transformation, where measurement error affects not only individual-level estimates but also the choice between group-level estimators. Hake's (1998) landmark study popularized ngains in physics education research to demonstrate the superiority of novel teaching methods over traditional approaches, and the measure has since gained traction in mathematics education research (Navarrete-Ulloa, 2025; Navarrete-Ulloa and Muñoz-Rubke, 2022).

The discrepancy between $\bar{ng}$ and $\hat{ng}$ reveals a progressive set of unresolved problems. To begin with, a systematic difference between the two estimates points to a possible bias in at least one of them—yet it is not clear which estimator, if either, can be trusted. This ambiguity is compounded by the absence of clear criteria for selecting one estimator over the other in practice. The root cause of both problems, we argue, is that no prior study has examined how measurement errors propagate through the computation of each estimator. We show that this omission is consequential: measurement errors render $\bar{ng}$ systematically biased while leaving $\hat{ng}$ asymptotically unbiased, directly explaining the observed discrepancies. Crucially, this result is not specific to ngains—it is a consequence of computing the mean of a nonlinear ratio from fallible measurements, a structure shared by many derived scores in educational and psychological research.

A second, related controversy concerns the validity of ngains as a measure of student growth. The ngain transformation was originally developed to control for the influence of initial knowledge on estimates of student growth. Support for this comes from studies showing that ngains and pretest scores are uncorrelated—that is, ngains remove the effect of prior knowledge (Hake, 1998, 2002; Coletta and Phillips, 2005; Coletta et al., 2007; Coletta and Steinert, 2020; Navarrete-Ulloa, 2025). Nevertheless, other studies have documented persistent correlations between pretest and ngain scores (Nissen et al., 2018), suggesting that ngains cannot fully control for prior knowledge and may favor certain subpopulations of learners. We contribute to this debate by showing that, even when student growth is entirely unaffected by prior knowledge, measurement errors alone can induce a systematic spurious correlation between pretest scores and ngains. This mechanism—spurious correlation as an artifact of measurement error in a nonlinear derived score—is not unique to ngains, and the framework we develop applies broadly to analogous metrics in educational and psychological measurement.

In summary, this study shows that measurement errors are one common cause behind both the estimator discrepancy and the pretest-ngain correlation, and that neither phenomenon should be taken as evidence of a fundamental flaw in the ngain transformation. These findings pave the way for more accurate interpretations of student growth based on ngains and the development of novel methods to correct bias in ngain estimates.

The remainder of this paper is organized as follows. The remainder of this section presents the historical and empirical challenges faced by ngains. Section 2 reviews the ngain transformation and its associated controversies in depth, and introduces the Statistical Framework underlying our analysis. Section 3 presents the Results, organized in three parts: the theoretical properties of $\bar{ng}$ and $\hat{ng}$ under error-free measurement conditions (Section 3.1), analogous results when measurement errors are present (Section 3.2), and computational simulations that validate all theoretical findings (Section 3.3). Section 4 discusses the implications for educational research, provides practical recommendations for using the two estimators, and outlines directions for future research. Section 5 concludes.

### 1.1 Controversies associated with normalized gains

Despite the widespread use of ngains in educational research (Hake, 1998, 2002; Nissen et al., 2018; Marx and Cummings, 2007; Bao, 2006; Coletta and Phillips, 2005; Coletta et al., 2007; Coletta and Steinert, 2020; Navarrete-Ulloa, 2025), this data transformation faces significant conceptual challenges related to its interpretation, estimation, and application as a measure of student growth. This section provides the historical and empirical background needed to understand these challenges and their implications.

#### 1.1.1 The criterion of pretest independence

Early research established that “optimal measures of knowledge change” should not correlate with pretest scores (O'Connor, 1972), since such correlations indicate an inability to compare student growth fairly across different levels of prior knowledge. Substantial evidence shows that raw gains (posttest–pretest) correlate strongly with pretest scores, challenging their validity: raw gains favor certain subgroups of learners and fail to adequately correct for the impact of prior knowledge. Recent research has proposed that ngains address this limitation by functioning as a correction of raw gains that controls for the effect of initial knowledge (Navarrete-Ulloa, 2025). The formal definitions of the individual ngain and the two group-level estimators are presented in Section 2; here we focus on the conceptual and empirical debates surrounding them.

#### 1.1.2 The pretest-ngain correlation debate

Although ngains were designed to be independent of pretest scores, several studies have reported persistent pretest-ngain correlations (Nissen et al., 2018), suggesting that ngains cannot fully control for prior knowledge and may favor certain subpopulations of learners. Others defend the validity of ngains by arguing that these correlations may result from methodological flaws, such as a poorly designed study or the omission of key control variables (Coletta and Steinert, 2020). The present study offers a complementary explanation: observed pretest-ngain correlations can be induced by measurement errors in pretest and posttest scores and should therefore not be interpreted as direct evidence of an intrinsic bias in the ngain transformation.

#### 1.1.3 The estimator discrepancy and Bao's statement

The pretest-ngain correlation has also been linked to the difference between the two group-level estimators ($\bar{ng}--\hat{ng}$). In particular, Bao (2006) argued that this difference is informative about the learning process:

“if $\bar{ng}$ is greater than $\hat{ng}$, students with low pretest scores tend to have smaller or similar score improvements compared to those with high pretest scores. Conversely, if $\bar{ng}$ is less than $\hat{ng}$, students with low pretest scores tend to show larger improvements than those with high pretest scores.”

In more formal terms, this relationship can be summarized as:


$$\begin{array}{l} \bar{ng}\leq \hat{ng}\text{ if and only if }\text{pretest-ngain correlation is negative}\\ \text{or zero.} \end{array}$$ (1)

This characterization implies that $\bar{ng}=\hat{ng}$ is equivalent to a zero pretest-ngain correlation—and thus that the ngain estimator controls for prior knowledge only when equality holds. Such a conclusion can be misleading, however, because it does not account for measurement errors. In this study, we show that measurement errors are linked to both phenomena simultaneously: the non-zero difference $\left(\bar{ng}-\hat{ng}\right)$ and the non-zero pretest-ngain correlation. More precisely, we confirm that statement (Equation 1) holds, and that the pretest-ngain correlation is always negative in the presence of errors—but our interpretation is fundamentally different from Bao's. The discrepancy arises because $\bar{ng}$ is biased and systematically lower than $\hat{ng}$, which is asymptotically unbiased. The difference between the estimators reflects spurious noise, not a genuine feature of the learning process.

#### 1.1.4 The bias-flexibility trade-off

A final, practical dimension of the controversy concerns the relative utility of the two estimators. The estimator $\bar{ng}$ allows researchers to model growth as a function of individual variables—such as age, cognitive abilities, socioeconomic status, and teaching methods—making it compatible with techniques such as ANCOVA and linear mixed models. In contrast, $\hat{ng}$ has more limited practical utility because it cannot incorporate individual-level information. This study establishes a fundamental trade-off: $\hat{ng}$ is the more accurate estimator, while $\bar{ng}$ is the more flexible one. Understanding this trade-off is essential for choosing the appropriate estimator and interpreting results correctly in educational research.

## 2 Materials and methods: statistical framework

### 2.1 Normalized gain: definitions

We assume that pretest and posttest measures are parallel and that *M* is the maximum possible score attainable in each. For each student *i*, the individual normalized gain (*ng*_*i*_) is defined as:


$$\begin{array}{l} ng_{i}=\frac{{\text{posttest}}_{i}-{\text{pretest}}_{i}}{M-{\text{pretest}}_{i}}. \end{array}$$ (2)

The first group-level estimation method computes the average ngain of the group ($\bar{ng}$):


$$\begin{array}{l} \bar{ng}=\frac{1}{N}\overset{N}{\underset{i=1}{\sum }}ng_{i}. \end{array}$$ (3)

The second method computes the “ngain of the average learner” ($\hat{ng}$):


$$\begin{array}{l} \hat{ng}=\frac{\frac{1}{N}\sum_{i=1}^{N}{\text{posttest}}_{i}-\frac{1}{N}\sum_{i=1}^{N}{\text{pretest}}_{i}}{M-\frac{1}{N}\sum_{i=1}^{N}{\text{pretest}}_{i}}. \end{array}$$ (4)

The estimators in Equations 3 and 4 are the empirical quantities studied throughout this paper. Their theoretical properties are analyzed via the Learning Rate Model introduced below.

### 2.2 The learning rate model

The Learning Rate Model (LR-Model) was recently introduced (Navarrete-Ulloa, 2025) as a theoretical framework to explain normalized gain scores as defined in Equation 2. In that work it was also applied to two empirical mathematics-learning datasets, providing initial support for its use with real data. The model assumes that the knowledge delivered through instruction is bounded and that learners' pretest (*X*) and posttest (*Y*) scores are measured without error by means of parallel tests. It additionally assumes 0 ≤ *X* ≤ *Y* ≤ 1 and *X* < 1 (with probability 1). For simplicity, the maximum achievable score *M* in Equation 2 is standardized to 1 (representing 100% achievement).

The LR-Model posits that the final knowledge status *Y* is determined by two independent random variables: the initial knowledge status *X* and the learning rate *F*. The independence of *X* and *F* is a core assumption of the model, reflecting the idea that a learner's capacity to acquire new knowledge under a given instructional condition should not depend on how much they already know at the outset. This assumption is what allows ngains to serve as a measure of student growth that is, in principle, free from the influence of prior knowledge. Under this assumption, the raw gain *G* = *Y*−*X* is proportional to both the amount of unfamiliar knowledge available to the learner (1−*X*) and the learning rate *F*. Mathematically:


$$\begin{array}{l} Y=X+\left(1-X\right)F. \end{array}$$ (5)

Notably, the LR-Model predicts a negative correlation between pretest scores and raw gains whenever *F*>0 (Navarrete-Ulloa, 2025), providing a theoretical rationale for an empirical regularity that early researchers (Cronbach and Furby, 1970; O'Connor, 1972) had treated as anomalous or attributed to measurement error.

The LR-Model is deliberately parsimonious: it summarizes pre-to-post change by a single scalar *F* and abstracts away from learning dynamics, forgetting (it assumes *Y*≥*X*), item difficulty and structure, and transfer—features that richer trajectory and growth-curve models (Liu and Perera, 2024; Commins et al., 2024) represent explicitly. Our aim is not to model these processes but to characterize the group-level estimators of μ_*F*_ implied by the ngain transformation.

Here, the learning rate *F* represents the learner's ability to acquire unfamiliar knowledge under specific instructional conditions. The learning rate is a theoretical construct that can be empirically estimated using either $\bar{ng}$ or $\hat{ng}$. Two clarifications are in order. The label *rate* denotes a static proportion of the unlearned material (1−*X*) acquired between pretest and posttest, not a temporal rate of change as in dynamic learning models; we keep the term for continuity with Navarrete-Ulloa (2025) but caution against a time-dynamic reading. Moreover, because *X* is a proportion of the instrument's maximum score rather than of latent knowledge, *F* and μ_*F*_ are properties of the learner jointly with the instrument and the population, not transportable traits of the learner alone. The expected value of *F*, denoted μ_*F*_, can be derived by taking the expectation of both sides of Equation 5:


$$\begin{array}{l} F=\frac{Y-X}{1-X}, \end{array}$$ (6)

$$\begin{array}{l} \mu_{F}=\frac{\mu_{Y}-\mu_{X}}{1-\mu_{X}}, \end{array}$$ (7)

where μ_*X*_ and μ_*Y*_ are the expected values of *X* and *Y*, respectively.

To model the learning process of a group of students, we assume *n* pairs of independent and identically distributed random variables {(*X*_1_, *F*_1_), (*X*_2_, *F*_2_), …, (*X*_*n*_, *F*_*n*_)}. The posttest scores {*Y*_1_, *Y*_2_, …, *Y*_*n*_} are determined by Equation 5. Within this framework, we define two estimators of the mean learning rate μ_*F*_:


$$\begin{array}{l} \bar{F}=\frac{1}{n}\overset{n}{\underset{i=1}{\sum }}\frac{Y_{i}-X_{i}}{1-X_{i}}, \end{array}$$ (8)

$$\begin{array}{l} \hat{F}=\frac{\frac{1}{n}\sum_{i=1}^{n}Y_{i}-\frac{1}{n}\sum_{i=1}^{n}X_{i}}{1-\frac{1}{n}\sum_{i=1}^{n}X_{i}}. \end{array}$$ (9)

Here, $\bar{F}$ and $\hat{F}$ are the theoretical counterparts of $\bar{ng}$ and $\hat{ng}$, respectively. As shown in Sections 3.1 and 3.2, in the absence of measurement errors both estimators are unbiased, while in the presence of measurement errors $\hat{F}$ remains asymptotically unbiased whereas $\bar{F}$ acquires a systematic negative bias.

### 2.3 The measurement error assumption

In psychometrics, a standard assumption is that a test measures a true latent variable plus an additive error (Kenneth and Whitney, 2005). To incorporate measurement errors into our framework, we define a random variable analogous to Equation 6 but accounting for errors in pretest and posttest scores. Let ϵ_*X*_ and ϵ_*Y*_ denote the measurement errors for the pretest and posttest, respectively. We assume these errors have zero means and are independent of all other random variables. We further assume that the pretest error distribution respects the admissibility of the observed scores, so that X^*^ < 1 almost surely and *E*[(1−^X^*^)−1^] < ∞. This condition holds whenever the errors are bounded—for instance, because observed scores are confined to the admissible range [0, 1]—and guarantees both that the noisy learning rate F^*^ in Equation 10 is well-defined and that its first moment exists. Additive errors with unbounded support (such as exact Gaussian errors) formally violate this condition, since *E*[(1−^X^*^)−1^] then diverges; in the simulations of Section 3.3 this is handled by restricting attention to admissible draws (see Table 1). Crucially, external observers have access only to the noisy measurements:


$$\begin{array}{l} X^{*}=X+\epsilon_{X},\\ Y^{*}=Y+\epsilon_{Y}, \end{array}$$

and consequently, the observed (noisy) learning rate F^*^ is defined as:


$$\begin{array}{l} F^{*}=\frac{Y^{*}-X^{*}}{1-X^{*}}. \end{array}$$ (10)

**Table 1:** Discarded observations in the computational simulation, by pretest reliability $\rho_{X^{*}}^{2}$.

		Discards per	Samples with ≥1 discard (%)		
Reliability $\rho_{X^{*}}^{2}$	Discard rate (%)	10, 000 learners	*n* = 30	*n* = 100	*n* = 500
0.70	0.0306	3.06	0.92	3.02	14.21
0.75	0.0089	0.89	0.27	0.89	4.35
0.80	0.0016	0.16	0.05	0.16	0.80
0.85	0.0002	0.02	0.01	0.02	0.10
0.90	< 0.0001	< 0.01	< 0.01	< 0.01	0.01
0.95	0.0000	0.00	0.00	0.00	0.00
1.00	0.0000	0.00	0.00	0.00	0.00

An observation is discarded when its noisy learning rate falls outside the admissible range, F^*^∉[0, 1]. Rates are computed over 2 × 10^7^ simulated learners and are essentially invariant to the sample size *n*. Every discard corresponds to a noise-induced negative gain (Y^*^<X^*^, i.e. F^*^ < 0); overshoot (Y^*^>1) and invalid pretest (X^*^≥1) discards did not occur. The last three columns report the percentage of samples losing at least one learner, which grows with *n* even though the overall fraction of discarded data remains negligible.

### 2.4 Summary of the framework

The statistical framework established in this section provides all the tools needed for the analyses that follow. The LR-Model specifies how true pretest scores *X* and true learning rates *F* jointly determine posttest scores *Y* via Equation 5, under the key assumption that *X*⊥*F*. The estimators $\bar{F}$ and $\hat{F}$ translate this model into computable group-level quantities. The measurement error model then introduces observed counterparts X^*^, Y^*^, and F^*^, which are the quantities actually available to researchers. Sections 3.1 and 3.2 analyze how well $\bar{F}$ and $\hat{F}$ recover μ_*F*_ under each scenario.

## 3 Results

### 3.1 Error-free measurement assumption

Under the assumption that learning is accurately described by Equation 5 and that the pretest (*X*) and posttest (*Y*) scores are observed without measurement errors, we establish two key results. First, we show that $\bar{F}$ is an unbiased estimator of μ_*F*_. Second, we demonstrate that $\hat{F}$ is also an *exactly* unbiased and consistent estimator of μ_*F*_. In other words, in the absence of measurement errors, both $\bar{F}$ (Equation 8) and $\hat{F}$ (Equation 9) provide unbiased estimates of μ_*F*_.

#### 3.1.1 Analysis of $\bar{F}$

Since $\bar{F}=\frac{1}{n}\overset{n}{\underset{i=1}{\sum }}F_{i}$ and each *F*_*i*_ has mean μ_*F*_, linearity of expectation immediately gives:


$$\begin{array}{l} \text{𝔼}\left[\bar{F}\right]=\frac{1}{n}\overset{n}{\underset{i=1}{\sum }}\mu_{F}=\mu_{F}. \end{array}$$

Thus $\bar{F}$ is an unbiased estimator of μ_*F*_. Furthermore, by the central limit theorem, $\bar{F}$ is also a consistent estimator of μ_*F*_.

#### 3.1.2 Analysis of $\hat{F}$

To analyze $\hat{F}$, we first derive the covariance matrix of (*X, Y*). From the LR-Model (Equation 5) and the independence of *X* and *F*, the computations in Navarrete-Ulloa (2025) allow us to infer:


$$\begin{array}{r} \text{Cov}\left(X,Y\right)=\text{Cov}\left(X,X+\left(1-X\right)F\right)=\sigma_{X}^{2}+\text{Cov}\left(X,\left(1-X\right)F\right)\\ =\sigma_{X}^{2}+\mu_{F}\text{Cov}\left(X,1-X\right)=\sigma_{X}^{2}-\mu_{F}\sigma_{X}^{2}=\left(1-\mu_{F}\right)\sigma_{X}^{2}, \end{array}$$

where the second equality uses the independence of *F* from *X*, so that Cov(*X*, (1−*X*)*F*) = μ_*F*_Cov(*X*, 1−*X*), and the last line uses $\text{Cov}\left(X,1-X\right)=-\sigma_{X}^{2}$. so the covariance matrix of (*X, Y*) is:


$$\begin{array}{l} \text{Σ}=\left[\begin{array}{cc} \sigma_{X}^{2} & \left(1-\mu_{F}\right)\sigma_{X}^{2}\\ \left(1-\mu_{F}\right)\sigma_{X}^{2} & \sigma_{Y}^{2} \end{array}\right]. \end{array}$$

By the central limit theorem, the sample means $\bar{X}$ and $\bar{Y}$ satisfy:


$$\begin{array}{l} \sqrt{n}\left(\left(\begin{array}{c} \bar{X}\\ Ȳ \end{array}\right)-\left(\begin{array}{c} \mu_{X}\\ \mu_{Y} \end{array}\right)\right)\to N\left(\left(\begin{array}{c} 0\\ 0 \end{array}\right),\text{Σ}\right). \end{array}$$ (11)

Next, we observe that $\hat{F}$ can be expressed as a function of $\bar{X}$ and $\bar{Y}$:


$$\begin{array}{l} \hat{F}=f\left(\bar{X},Ȳ\right),\;\text{where }f\left(x,y\right)=\frac{y-x}{1-x}. \end{array}$$

Since *f*(μ_*X*_, μ_*Y*_) = μ_*F*_ (from Equation 7), applying the delta method in Equation 11 yields:


$$\begin{array}{l} \sqrt{n}\left(\hat{F}-\mu_{F}\right)\to N\left(0,\sigma_{\hat{F}}^{2}\right), \end{array}$$ (12)

the asymptotic variance $\sigma_{\hat{F}}^{2}$ is given by in Equation 12:


$$\begin{array}{l} \sigma_{\hat{F}}^{2}=\frac{\sigma_{Y}^{2}{\left(1-\mu_{X}\right)}^{2}-\sigma_{X}^{2}{\left(1-\mu_{Y}\right)}^{2}}{{\left(1-\mu_{X}\right)}^{4}}. \end{array}$$

This result implies that $\hat{F}$ is a consistent estimator of μ_*F*_. Moreover, $\hat{F}$ is *exactly* unbiased: applying the tower property of conditional expectation, and since each *F*_*i*_ is independent of *X*_1_, *X*_2_, …, *X*_*n*_ with *X*_*i*_ < 1, we have:


$$\begin{array}{l} E[\hat{F}]=E\left[\frac{\frac{1}{n}\sum_{i=1}^{n}\left(Y_{i}-X_{i}\right)}{\frac{1}{n}\sum_{i=1}^{n}\left(1-X_{i}\right)}\right]\\ \;=E\left[\frac{\sum_{i=1}^{n}F_{i}(1-X_{i})}{\sum_{i=1}^{n}\left(1-X_{i}\right)}\right]\\ \;=E\left[E\left[\frac{F_{1}(1-X_{1})}{\sum_{i=1}^{n}\left(1-X_{i}\right)}+\ldots +\frac{F_{n}(1-X_{n})}{\sum_{i=1}^{n}\left(1-X_{i}\right)}|X_{1},\ldots ,X_{n}\right]\right]\\ \;=E\left[\frac{\mu_{F}(1-X_{1})}{\sum_{i=1}^{n}\left(1-X_{i}\right)}+\ldots +\frac{\mu_{F}(1-X_{n})}{\sum_{i=1}^{n}\left(1-X_{i}\right)}\right]\\ \;=\mu_{F}. \end{array}$$

#### 3.1.3 Relative efficiency of $\bar{F}$ with respect to $\hat{F}$

Since $\bar{F}$ and $\hat{F}$ are both unbiased, their relative efficiency can be compared through their asymptotic variances. Developing $\sigma_{Y}^{2}$ under the LR-Model with *X*⊥*F*, the asymptotic variance of $\hat{F}$ admits the closed form


$$\begin{array}{l} \sigma_{\hat{F}}^{2}=\sigma_{F}^{2}\left(1+\frac{\sigma_{X}^{2}}{{\left(1-\mu_{X}\right)}^{2}}\right), \end{array}$$ (13)

where $\sigma_{F}^{2}=\text{Var}\left(F\right)$ is the variance of the learning rate. This same quantity is the asymptotic variance of $\bar{F}$: since $\bar{F}=\frac{1}{n}\overset{n}{\underset{i=1}{\sum }}F_{i}$ is a sample mean of i.i.d. terms, $\sqrt{n}\left(\bar{F}-\mu_{F}\right)\to N\left(0,\sigma_{F}^{2}\right)$, equivalently $n\text{Var}\left(\bar{F}\right)=\sigma_{F}^{2}$. Hence, from Equation 13 follows that the two estimators can be compared on the same scale, and


$$\begin{array}{l} \sigma_{\hat{F}}^{2}-\sigma_{F}^{2}=\sigma_{F}^{2}\frac{\sigma_{X}^{2}}{{\left(1-\mu_{X}\right)}^{2}}\geq 0, \end{array}$$ (14)

so $\bar{F}$ is always at least as asymptotically efficient as $\hat{F}$, with equality if and only if $\sigma_{X}^{2}=0$. Equation 14 shows that the efficiency gap is governed by the dispersion of the *pretest* scores relative to the ceiling, $\sigma_{X}^{2}/{\left(1-\mu_{X}\right)}^{2}$, not by the heterogeneity of the learning rate: when pretest scores are widely spread, aggregating them before forming the ratio (as $\hat{F}$ does) discards more information, making $\bar{F}$ the more efficient estimator in the error-free case.

#### 3.1.4 Summary

Both $\bar{F}$ and $\hat{F}$ are unbiased estimators of μ_*F*_. Any observed differences between them are due solely to random variation. This analysis provides a theoretical foundation for understanding the behavior of these estimators under ideal conditions.

### 3.2 Measurement errors assumption

#### 3.2.1 Estimators for μ_*F*_

Consider *n* pairs of independent and identically distributed random variables ${\left\{\left(X_{i},F_{i}\right)\right\}}_{i=1}^{n}$, with posttest scores ${\left\{Y_{i}\right\}}_{i=1}^{n}$ determined by Equation 5. Let ${\left\{\left(\epsilon_{X_{i}},\epsilon_{Y_{i}}\right)\right\}}_{i=1}^{n}$ denote the corresponding measurement errors, leading to observed scores ${\left\{\left(X_{i}^{*},Y_{i}^{*}\right)\right\}}_{i=1}^{n}$. The observed learning rates ${\left\{F_{i}^{*}\right\}}_{i=1}^{n}$ are obtained using Equation 10.

We define two estimators of μ_*F*_ based on the noisy observations:


$$\begin{array}{l} \bar{F^{*}}=\frac{1}{n}\overset{n}{\underset{i=1}{\sum }}\frac{Y_{i}^{*}-X_{i}^{*}}{1-X_{i}^{*}}=\frac{1}{n}\overset{n}{\underset{i=1}{\sum }}F_{i}^{*} \end{array}$$ (15)

$$\begin{array}{l} \hat{F^{*}}=\frac{\frac{1}{n}\sum_{i=1}^{n}Y_{i}^{*}-\frac{1}{n}\sum_{i=1}^{n}X_{i}^{*}}{1-\frac{1}{n}\sum_{i=1}^{n}X_{i}^{*}} \end{array}$$ (16)

The estimators in Equations 15 and 16 are the theoretical counterparts of $\bar{ng}$ and $\hat{ng}$. In the following subsections, we show that:

- $\hat{F^{*}}$ is a consistent (hence, under bounded errors, asymptotically unbiased) estimator of μ_*F*_.
- $\bar{F^{*}}$ is a biased estimator of μ_*F*_.

As a consequence, we offer a reinterpretation of Bao's statement (Equation 1): the difference between $\bar{F^{*}}$ and $\hat{F^{*}}$ reflects the bias of $\bar{F^{*}}$ rather than a genuine feature of the learning process. We also establish a formal connection between the bias of $\bar{F^{*}}$ and the correlation between the noisy pretest scores X^*^ and the noisy learning rates F^*^.

#### 3.2.2 Preliminary result: expected value of F^*^

Before establishing the bias of $\bar{F^{*}}$, we derive a general expression for *E*(F^*^) that will be used throughout this section. We begin with the identity:


$$\begin{array}{l} F^{*}\left(1-X^{*}\right)=Y^{*}-X^{*}. \end{array}$$

Taking the expected value of both sides and rearranging terms yields:


$$\begin{array}{l} \text{𝔼}\left(Y-X\right)=E\left(F^{*}\right)E\left(1-X\right)+\text{Cov}\left(F^{*},1-X^{*}\right). \end{array}$$

Solving for *E*(F^*^):


$$\begin{array}{l} \text{𝔼}\left(F^{*}\right)=\mu_{F}-\frac{\text{Cov}\left(F^{*},1-X^{*}\right)}{E\left(1-X\right)}\\ \;=\mu_{F}+\frac{\text{Cov}\left(F^{*},X^{*}\right)}{E\left(1-X\right)}\\ \;=\mu_{F}+\frac{\text{Cov}\left(F^{*},X^{*}\right)}{1-\mu_{X}}\\ \;=\mu_{F}+\sigma_{X^{*}}\sigma_{F^{*}}\frac{\text{corr}\left(F^{*},X^{*}\right)}{1-\mu_{X}}. \end{array}$$ (17)

Although *X* and *F* are independent (i.e., Cov(*X, F*) = 0), this does not imply that Cov(X, F^*^) = 0 or Cov(X^*^, F^*^) = 0. Since F^*^ depends on X^*^ (which includes ϵ_*X*_), F^*^ is statistically dependent on X^*^. Consequently, *E*(F^*^) deviates from μ_*F*_ by a term governed by the covariance between F^*^ and X^*^, which is itself induced by measurement error.

#### 3.2.3 Bias of $\bar{F}$^*^

We begin by showing that $\bar{F^{*}}$ is a biased estimator of μ_*F*_. By the central limit theorem, we have:


$$\sqrt{n}\left(\bar{F^{*}}-E(F^{*})\right)\to N\left(0,\sigma_{F^{*}}^{2}\right),\;\bar{F^{*}}\overset{p}{\to }E(F^{*})\neq \mu_{F}.$$

Using linearity and the preliminary result (Equation 17), we obtain the expected value of $\bar{F^{*}}$:


$$\begin{array}{l} \text{𝔼}\left(\bar{F^{*}}\right)=E\left(F^{*}\right)\\ \;=\mu_{F}+\frac{\text{Cov}\left(F^{*},X^{*}\right)}{E\left(1-X\right)}\\ \;=\mu_{F}+\sigma_{X^{*}}\sigma_{F^{*}}\frac{\text{corr}\left(F^{*},X^{*}\right)}{1-\mu_{X}}. \end{array}$$ (18)

This result shows that $\bar{F^{*}}$ is biased, with bias $\frac{\text{Cov}\left(F^{*},X^{*}\right)}{1-\mu_{X}}$, regardless of the sample size *n*. To determine the *sign* of this bias, we derive an exact expression for *E*(F^*^). Splitting the numerator of F^*^ as Y^*^−X^*^ = (Y^*^−1) + (1−X^*^) gives the identity


$$\begin{array}{l} F^{*}=\frac{Y^{*}-X^{*}}{1-X^{*}}=\frac{Y^{*}-1}{1-X^{*}}+1. \end{array}$$ (19)

The posttest measurement error ϵ_*Y*_ has zero mean and is independent of X^*^, so $\text{𝔼}\left[\epsilon_{Y}{\left(1-X^{*}\right)}^{-1}\right]=\text{𝔼}\left(\epsilon_{Y}\right)\text{𝔼}\left[{\left(1-X^{*}\right)}^{-1}\right]=0$, and we may replace Y^*^ by *Y* inside the expectation. Using the LR-Model relation *Y*−1 = −(1−*X*)(1−*F*) together with the independence of *F* from (X, X^*^), Equation 19 yields


$$\begin{array}{l} \text{𝔼}\left(F^{*}\right)=1+\text{𝔼}\left[\frac{Y-1}{1-X^{*}}\right]=1-\text{𝔼}\left[\frac{\left(1-X\right)\left(1-F\right)}{1-X^{*}}\right]\\ \;=1-\left(1-\mu_{F}\right)\text{𝔼}\left[\frac{1-X}{1-X^{*}}\right]. \end{array}$$ (20)

Subtracting μ_*F*_ from both sides of Equation 20 expresses the bias in factored form:


$$\begin{array}{l} \text{bias}\left(\bar{F^{*}}\right)=\text{𝔼}\left(F^{*}\right)-\mu_{F}=\left(1-\mu_{F}\right)\left(1-\text{𝔼}\left[\frac{1-X}{1-X^{*}}\right]\right). \end{array}$$ (21)

It remains to sign the factor 1−*E*[(1−*X*)/(1−X^*^)]. Write $1-X^{*}=\left(1-X\right)-\epsilon_{X}$, and condition on *X*. Under the admissibility assumption, 1−X^*^>0 almost surely, so ϵ_*X*_ < 1−*X* almost surely and the map *t*↦((1−*X*)−*t*)^−1^ is convex on the support of ϵ_*X*_. Since ϵ_*X*_ has zero mean and is independent of *X*, Jensen's inequality gives


$$\begin{array}{c} \text{𝔼}\left[\frac{1-X}{1-X^{*}}[X\right]=\left(1-X\right)\text{𝔼}\left[\frac{1}{\left(1-X\right)-\epsilon_{X}}]X\right]\\ \geq \left(1-X\right)\cdot \frac{1}{1-X}=1. \end{array}$$

Taking expectations over *X* preserves the inequality, so


$$\begin{array}{l} \text{𝔼}\left[\frac{1-X}{1-X^{*}}\right]\geq 1. \end{array}$$ (22)

Because 1−μ_*F*_>0, combining Equations 21, 22 shows that $\bar{F^{*}}$ exhibits a systematic negative bias in the presence of pretest measurement error:


$$\begin{array}{l} \text{bias}\left(\bar{F^{*}}\right)=\text{𝔼}\left(F^{*}\right)-\mu_{F}=\left(1-\mu_{F}\right)\left(1-\text{𝔼}\left[\frac{1-X}{1-X^{*}}\right]\right)\leq 0, \end{array}$$ (23)

with equality if and only if ϵ_*X*_ = 0 almost surely, that is., when the pretest is measured without error.

#### 3.2.4 Consistency and asymptotic unbiasedness of $\hat{F}$^*^

Next, we show that $\hat{F^{*}}$ is an asymptotically unbiased estimator of μ_*F*_. Since the measurement errors have zero mean and are mutually uncorrelated, the central limit theorem guarantees:


$$\begin{array}{l} \sqrt{n}\left(\left(\begin{array}{c} \bar{X^{*}}\\ \bar{Y^{*}} \end{array}\right)-\left(\begin{array}{c} \mu_{X}\\ \mu_{Y} \end{array}\right)\right)\to N\left(\left(\begin{array}{c} 0\\ 0 \end{array}\right),\text{Σ}+\left[\begin{array}{cc} \sigma_{\epsilon_{X}}^{2} & 0\\ 0 & \sigma_{\epsilon_{Y}}^{2} \end{array}\right]\right), \end{array}$$

where Σ is the covariance matrix of $\left(\begin{array}{c} X\\ Y \end{array}\right)$ and $\sigma_{\epsilon_{X}}^{2}$, $\sigma_{\epsilon_{Y}}^{2}$ are the variances of the measurement errors ϵ_*X*_ and ϵ_*Y*_, respectively.

The function $f\left(x,y\right)=\frac{y-x}{1-x}$ is continuously differentiable for 0 < *x*<*y* < 1 with gradient:


$$\begin{array}{l} \nabla f\left(x,y\right)=\left(\begin{array}{c} \frac{y-1}{{\left(1-x\right)}^{2}}\\ \frac{1}{1-x} \end{array}\right). \end{array}$$

Since *f*(μ_*X*_, μ_*Y*_) = μ_*F*_, the delta method (Shao, 2003) yields:


$$\begin{array}{l} \sqrt{n}\left(\hat{F^{*}}-\mu_{F}\right)\to N\left(0,\sigma_{\hat{F^{*}}}^{2}\right), \end{array}$$ (24)

where


$$\begin{array}{l} \sigma_{\hat{F^{*}}}^{2}=\frac{{\left(1-\text{𝔼}\left(Y\right)\right)}^{2}\left(\sigma_{\epsilon_{X}}^{2}-\sigma_{X}^{2}\right)+\left(\sigma_{Y}^{2}+\sigma_{\epsilon_{Y}}^{2}\right){\left(1-\text{𝔼}\left(X\right)\right)}^{2}}{{\left(1-\text{𝔼}\left(X\right)\right)}^{4}}<\infty , \end{array}$$

since *E*(*X*) < 1.

The convergence in Equation 24 implies in particular that $\hat{F^{*}}\overset{p}{\to }\mu_{F}$, since convergence in distribution to a degenerate (constant) limit is equivalent to convergence in probability (Billingsley, 2012). That is, $\hat{F^{*}}$ is a *consistent* estimator of μ_*F*_. Under the admissibility assumption of Section 3.2, the noisy learning rate is bounded, so $\hat{F^{*}}$ is uniformly integrable; consistency then yields $\text{𝔼}\left(\hat{F^{*}}\right)\to \mu_{F}$, and for sufficiently large *n*:


$$\begin{array}{l} \text{𝔼}\left(\hat{F^{*}}\right)\approx \mu_{F}. \end{array}$$

We emphasize, however, that this is an approximation, in contrast to the exact equality derived in Section 3.1.2 using the independence between pretest and ngain, which no longer holds under measurement errors.

#### 3.2.5 Bao's relationship between $\bar{F}$^*^ and $\hat{F}$^*^

To summarize, we have shown that $\text{𝔼}\left(\hat{F^{*}}\right)$ is close to μ_*F*_ for large *n*, while for $\bar{F^{*}}$ it holds (regardless of sample size) that:


$$\text{𝔼}\left(\bar{F^{*}}\right)=\mu_{F}+\frac{\text{Cov}\left(F^{*},X^{*}\right)}{\text{𝔼}\left(1-X\right)}.$$

Thus, for large *n*, the expected difference between the two estimators is:


$$\begin{array}{r} \text{𝔼}\left(\bar{F^{*}}\right)-\text{𝔼}\left(\hat{F^{*}}\right)\approx \frac{\text{Cov}\left(F^{*},X^{*}\right)}{\text{𝔼}\left(1-X\right)}\\ =\sigma_{F^{*}}\sigma_{X^{*}}\frac{\text{corr}\left(F^{*},X^{*}\right)}{\text{𝔼}\left(1-X\right)}, \end{array}$$

where corr(F^*^, X^*^) denotes the correlation between F^*^ and X^*^. In light of this result, Bao's statement (Equation 1) (Bao, 2006) can be restated as:


$$\begin{array}{l} \text{𝔼}\left[\bar{F^{*}}\right]\leq \text{𝔼}\left[\hat{F^{*}}\right]\;\text{if and only if}\;\text{corr}\left(F^{*},X^{*}\right)\leq 0. \end{array}$$ (25)

Equation 25, although formally similar to Equation 1, is compatible with the assumption of independence between true pretest scores and true learning rates, attributing the difference between the estimators entirely to measurement errors.

### 3.3 Computational simulations

In this section, we conduct computational simulations to validate the earlier theoretical results. Specifically, we compare the performance of the estimators $\bar{F^{*}}$ and $\hat{F^{*}}$, and illustrate the impact of measurement errors on the correlation between pretest scores and ngains. The simulations below reuse the same true scores across reliability levels, isolating the effect of measurement error on a common set of learners.

#### 3.3.1 Simulation setup

**Figure 1:**
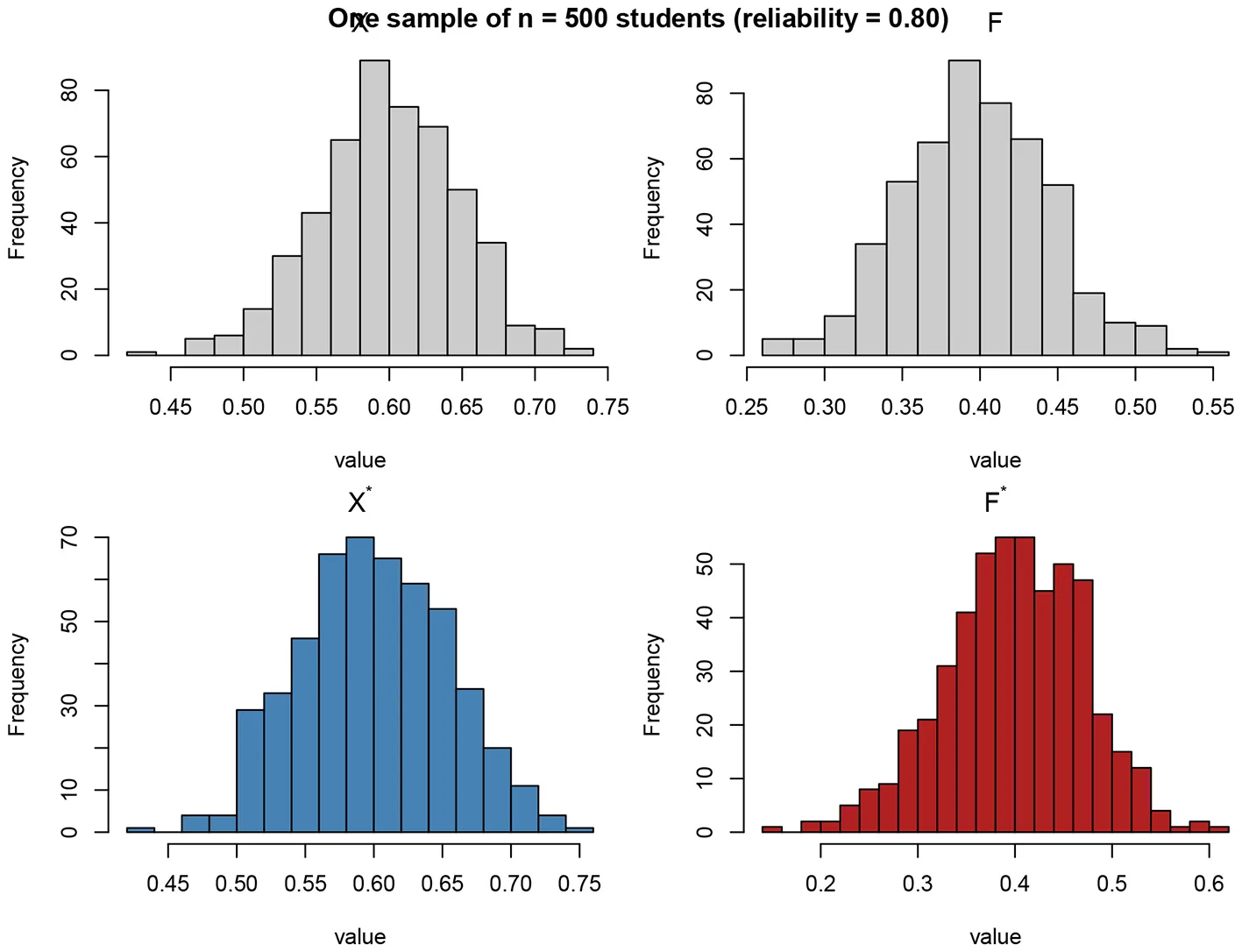
Simulation of *X*, *F*, X^*^, and F^*^ in one sample of 500 students.

We set sample sizes to *n* = 30, 100, 500, reflecting typical sample sizes in educational research. For each common set of learners, the true pretest scores *X* and learning rates *F* were generated from the beta distribution:


$$\begin{array}{l} X\sim \text{Beta}\left(\alpha_{X},\beta_{X}\right),\\ F\sim \text{Beta}\left(\alpha_{F},\beta_{F}\right), \end{array}$$

with parameters α_*X*_ = 60, β_*X*_ = 40, α_*F*_ = 40, and β_*F*_ = 60. These parameters were chosen to produce bell-shaped distributions that approximate real-world educational data (Navarrete-Ulloa, 2025). The beta distribution closely approximates a Gaussian distribution for large parameter values (Wise, 1960). Figure 1 shows the empirical distributions of the simulated values.

To introduce measurement errors, we varied the reliability of the pretest scores between 0.7 and 1.0, a range considered acceptable in educational measurement (Kenneth and Whitney, 2005). For each reliability level, we generated two independent vectors of zero-mean Gaussian errors, ϵ_*X*_ and ϵ_*Y*_, and defined the observed scores as:


$$\begin{array}{l} X^{*}=X+\epsilon_{X},\\ Y^{*}=X+F\left(1-X\right)+\epsilon_{Y}. \end{array}$$

The noisy learning rates F^*^ were then computed as F^*^ = (Y^*^−X^*^)/(1−X^*^), discarding any simulated value for which F^*^∉[0, 1]—that is, instances where Y^*^ ≤ X^*^ or X^*^≥1, which are inconsistent with the LR-Model (Navarrete-Ulloa, 2025). The reliability ρ^2^ of X^*^ is given by:


$$\begin{array}{l} \rho_{X^{*}}^{2}=\frac{\sigma_{X}^{2}}{\sigma_{X^{*}}^{2}}=1-\frac{\sigma_{\epsilon_{X}}^{2}}{\sigma_{X^{*}}^{2}}, \end{array}$$

where $\sigma_{\epsilon_{X}}^{2}$ is the variance of the pretest measurement error.

#### 3.3.2 Simulation results

For each sample size (*n* = 30, 100, and 500) and reliability level, we repeated the simulation procedure *N* = 1, 000 times to estimate the performance of $\bar{F^{*}}$ and $\hat{F^{*}}$ and to isolate the effect of measurement errors on the estimators by varying the noise level (reliability) while keeping the true *X* and *F* data constant across simulations.

**Figure 2:**
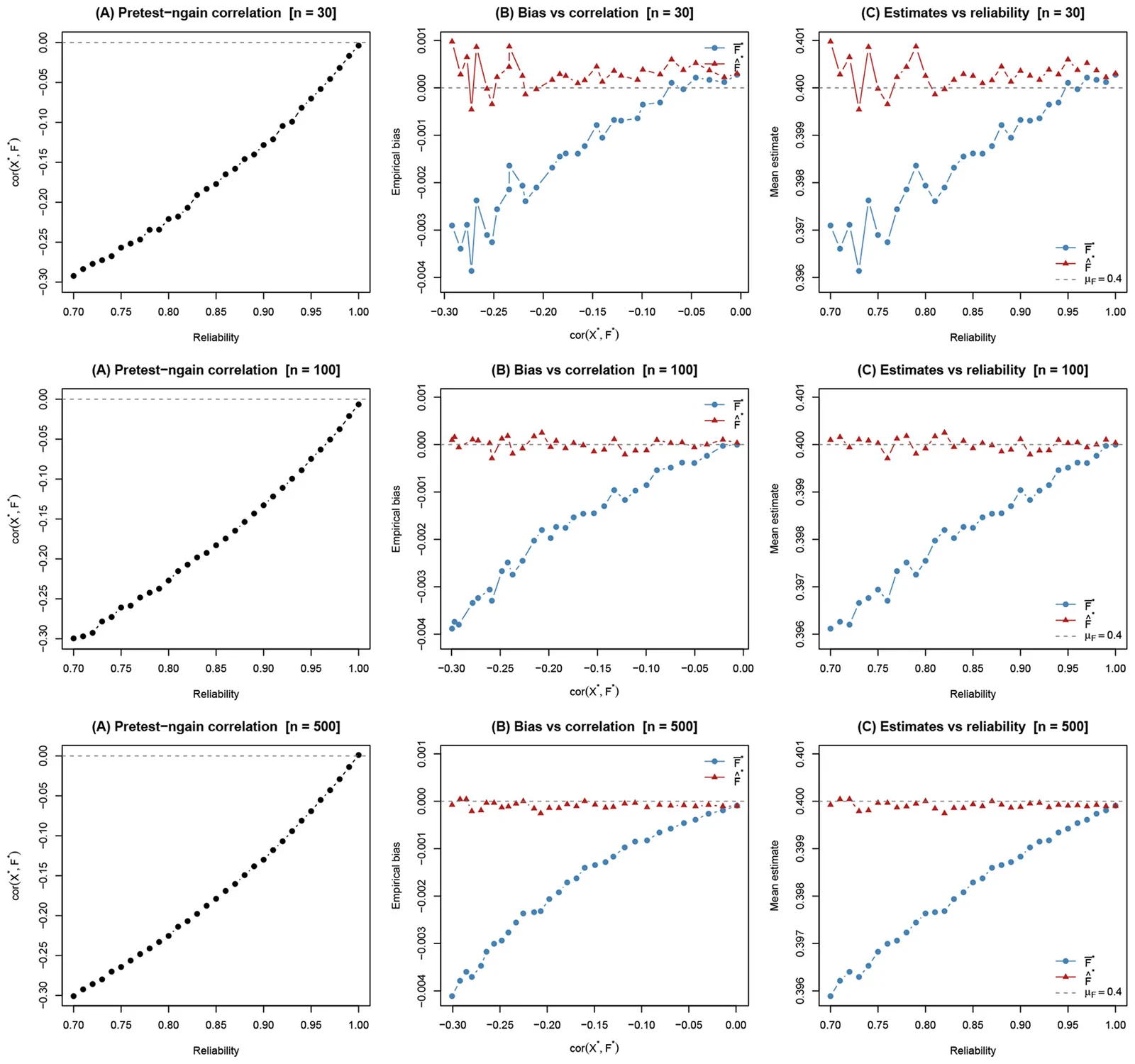
**(A)** Empirical correlation between noisy pretest scores (X^*^) and noisy learning rates (F^*^) as a function of reliability. **(B)** Empirical bias of $\bar{F^{*}}$ (blue) and $\hat{F^{*}}$ (red) as a function of the observed correlation. **(C)** Sample mean of estimates $\bar{F^{*}}$ (blue) and $\hat{F^{*}}$ (red) as functions of reliability.

The three panels of Figure 2 summarize the simulation results. Panel A shows that measurement errors in X^*^ induce a negative correlation between F^*^ and X^*^. Since *F* and *X* are independent by design, this correlation is purely an artifact of measurement errors. Panel B demonstrates that this spurious correlation introduces systematic bias in $\bar{F^{*}}$, while $\hat{F^{*}}$ remains unbiased, centered around the true value μ_*F*_ = 0.4. Panel C further illustrates that the bias in $\bar{F^{*}}$ decreases as reliability increases, whereas $\hat{F^{*}}$ remains stable across all reliability levels.

Table 1 shows that across all reliability levels and sample sizes, fewer than 0.1% of simulated learners' data were discarded (and none for reliability ≥0.85), almost all arising from noise-induced negative gains (Y^*^<X^*^), while violations from X^*^≥1 or Y^*^>1 were negligible. It is worth emphasizing however that these values are highly dependent on the parameters of the simulation presented here.

#### 3.3.3 Unconditional repeated-sampling assessment

The simulations above reuse the same true scores across reliability levels, isolating the effect of measurement error on a common set of learners. To complement this, we conducted a fully unconditional experiment in which new true pretest scores *X* and learning rates *F* are drawn independently in every repetition, for sample sizes *n* ∈ {30, 100, 500}. For each estimator we report its empirical bias (with 95% Monte Carlo confidence bands), variance, and mean squared error (MSE = Variance+Bias^2^) as functions of reliability.

**Figure 3:**
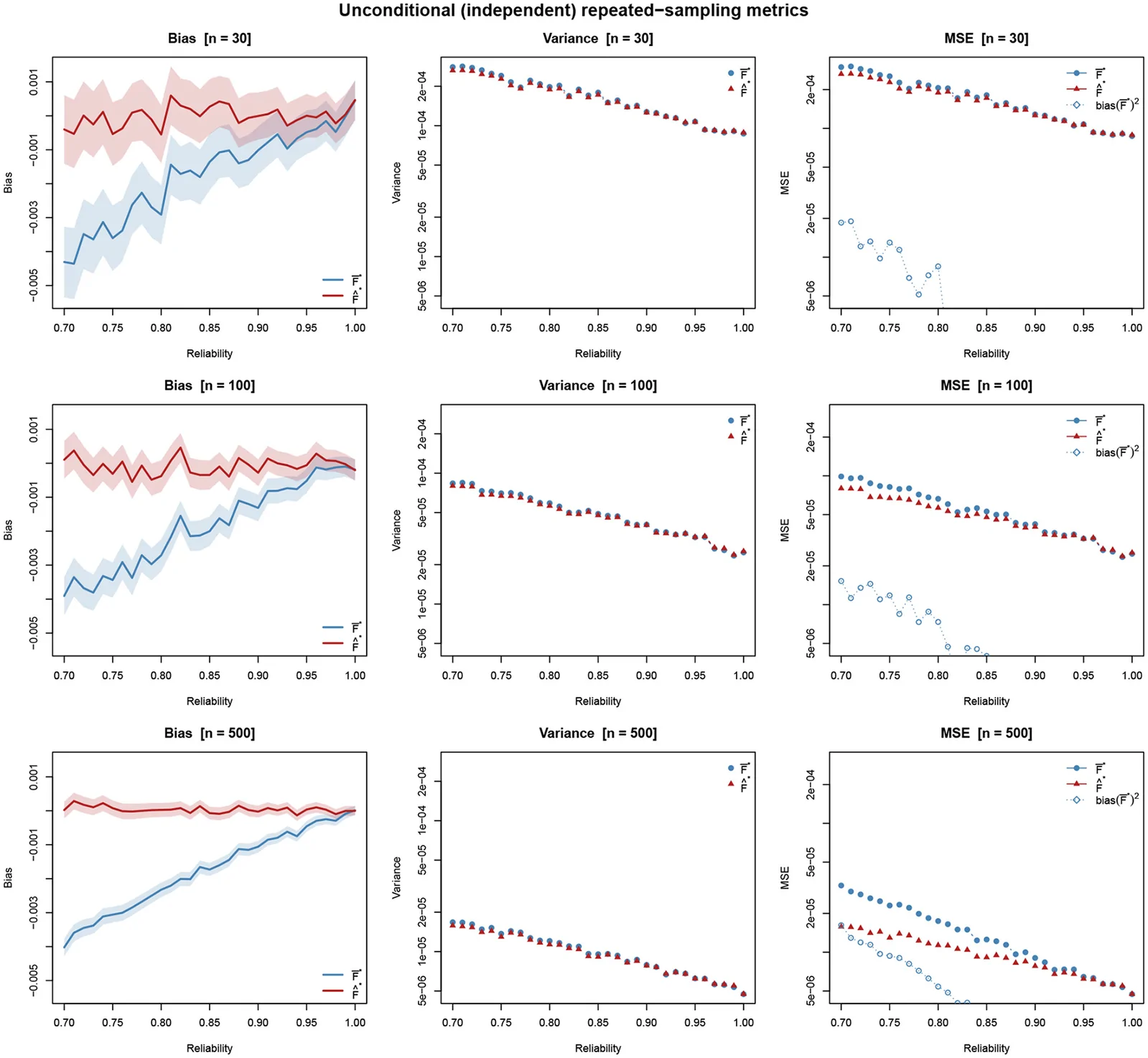
Unconditional repeated-sampling performance of $\bar{F^{*}}$ (blue) and $\hat{F^{*}}$ (red) as a function of pretest reliability, for *n* = 30 (top), *n* = 100 (middle), and *n* = 500 (bottom). Fresh true scores *X* and learning rates *F* are generated in each of the *R* = 1000 repetitions per cell. **(Left)** Empirical bias with 95% Monte Carlo confidence bands; the bias of $\bar{F^{*}}$ is negative and nearly invariant in *n*, while $\hat{F^{*}}$ is centered on μ_*F*_ = 0.4. **(Center)** Variance (log scale), decreasing at the 1/*n* rate for both estimators. **(Right)** Mean squared error (log scale); the MSE of $\hat{F^{*}}$ vanishes as *n* grows, whereas the MSE of $\bar{F^{*}}$ is bounded below by its squared bias (dotted line), demonstrating inconsistency under measurement error.

Figure 3 shows the outcome. The bias of $\bar{F^{*}}$ is essentially invariant in *n*—consistent with the sample-size independence established in Equation 18—and is negative whenever reliability is below one, whereas $\hat{F^{*}}$ stays centered on μ_*F*_ = 0.4 at every reliability level. The variance of both estimators decreases at the expected 1/*n* rate. Consequently their mean squared errors diverge in behavior: the MSE of $\hat{F^{*}}$ tends to zero as *n* grows, so $\hat{F^{*}}$ is consistent, while the MSE of $\bar{F^{*}}$ decreases only until it reaches the squared-bias floor $\text{bias}{\left(\bar{F^{*}}\right)}^{2}$ (dotted line) and cannot fall below it. Under measurement error, $\bar{F^{*}}$ is therefore inconsistent: no sample size removes its systematic negative bias, and for large *n* its error is dominated by that bias rather than by sampling variability.

#### 3.3.4 Key findings

** Remark 1 (Summary of simulation findings)**. The following patterns were observed consistently in both experiments—the conditional design (common true scores reused across reliability levels) and the unconditional design (fresh *X* and *F* drawn in every repetition)—across *R* = 1, 000 repetitions per cell:

1. **Bias of** $\bar{F^{*}}$**:** As reliability decreases, $\bar{F^{*}}$ exhibits an increasing negative bias, systematically underestimating μ_*F*_. This bias is essentially invariant to the sample size *n*, confirming the sample-size independence of the bias established in Equation 18.
2. **Unbiasedness of** $\bar{F^{*}}$**:** Across all reliability levels and all sample sizes, $\hat{F^{*}}$ remains centered on the true value μ_*F*_ = 0.4, with 95% Monte Carlo confidence bands that include zero bias throughout.
3. **Pretest–ngain correlation:** The empirical correlation between X^*^ and F^*^ decreases as a function of reliability. When measurement errors are negligible ($\rho_{X^{*}}^{2}\approx 1$) the correlation is nearly zero, and a stronger negative association emerges as reliability decreases. This supports our claim that the observed pretest–ngain correlation is an artifact of measurement error rather than an intrinsic property of the learning process.
4. **Consistency and mean squared error:** The variance of both estimators decreases at the 1/*n* rate. Consequently, $\hat{F^{*}}$ is consistent—its mean squared error tends to zero as *n* grows— whereas the mean squared error of $\bar{F^{*}}$ is bounded below by its squared bias and cannot vanish. Under measurement error, $\bar{F^{*}}$ is therefore inconsistent: no sample size removes its systematic negative bias, and for large *n* its error is dominated by bias rather than by sampling variability.
5. **Reinterpretation of Bao's statement:** The difference between $\bar{F^{*}}$ and $\hat{F^{*}}$ is explained by the bias of $\bar{F^{*}}$ induced by measurement error, which in turn produces a spurious correlation between pretest scores and ngains (see Equation 25).

The bias in $\bar{F^{*}}$ highlights the importance of accounting for measurement errors when estimating learning rates. While $\hat{F^{*}}$ is empirically robust to measurement errors for large samples, $\bar{F^{*}}$ can lead to misleading conclusions if left uncorrected. Our theoretical results (Equation 18) and simulations demonstrate that the pretest-ngain correlation directly governs the difference between $\bar{F^{*}}$ and $\hat{F^{*}}$, a finding that is crucial for interpreting learning data in educational research.

## 4 Discussion

The central finding of this study is that measurement error is one common cause behind two phenomena that have separately troubled the ngains literature for nearly two decades: the systematic discrepancy between $\bar{ng}$ and $\hat{ng}$, and the persistent correlation between pretest scores and ngains. By establishing that measurement error is one of the causes of both phenomena within a unified statistical framework, the present study clarifies what these phenomena mean—and, crucially, what they do not mean—for the interpretation of student growth in educational research. When measurement errors are absent, both $\bar{ng}$ and $\hat{ng}$ are unbiased estimators of the group learning rate μ_*F*_, and any observed differences between them are attributable solely to random variation. In the presence of measurement errors, however, the two estimators diverge systematically: $\hat{ng}$ remains asymptotically unbiased, while $\bar{ng}$ acquires a negative bias equal to Cov(F^*^, X^*^)/*E*(1−*X*) (Equation 18), regardless of sample size. This asymmetry establishes a fundamental bias-flexibility trade-off that researchers must navigate when choosing between the two estimators.

### 4.1 Reinterpreting Bao's Statement

One of the most precise contributions of this study is a reinterpretation of Bao's (2006) widely cited characterization of the learning process. Bao argued that the sign of $\left(\bar{ng}-\hat{ng}\right)$ reveals whether low-pretest students gain more or less than high-pretest students. Our results confirm the mathematical relationship in Equation 1 but attribute it to an entirely different cause: the bias of $\bar{ng}$ induced by measurement error, which simultaneously produces a spurious negative correlation between pretest scores and ngains (Equation 25). Since *F* and *X* are independent by assumption in the LR-Model, and our simulations confirm this independence holds by design, the sign of $\left(\bar{ng}-\hat{ng}\right)$ carries no information about how students at different pretest levels learn. It reflects measurement noise, not a property of the instructional process. Researchers who have interpreted this difference as a substantive psychological finding should therefore do so with considerable caution.

### 4.2 The pretest-ngain correlation debate

The claim that ngains are “biased in favor of high pretest populations” (Nissen et al., 2018) has generated significant controversy, as it appears to undermine the core purpose of the ngain transformation—controlling for the effect of initial knowledge. Our results offer a principled alternative explanation: observed pretest-ngain correlations may arise entirely from low reliability in pretest and posttest scores, with no implication of genuine dependence between prior knowledge and learning capacity. This interpretation is consistent with Coletta and Steinert's (2020) defense of ngains and provides a theoretical mechanism for the methodological flaws they invoked informally.

Importantly, our findings do not imply that pretest-ngain correlations never reflect genuine dependencies. Rather, they show that such correlations cannot be taken at face value without first accounting for measurement reliability. When reliability is high, a significant pretest-ngain correlation is a meaningful signal of uncontrolled variables or model misfit (Navarrete-Ulloa, 2025). When reliability is moderate to low, the same correlation may be entirely artifactual. This distinction reframes the debate from a question about the validity of ngains as a construct to a more tractable empirical question about the reliability of the measurement instruments used.

Another key element is the central hypothesis of the LR-Model (Navarrete-Ulloa, 2025): that the learning rate is independent of a student's initial knowledge. Because normalized gain is, by construction, a function of the pretest, this independence is a nontrivial claim rather than an artifact of the measure, and whether it holds depends on the specific learning phenomenon. It is therefore an empirical question—and one that much of the learning-sciences literature on expertise, schema formation, and Matthew effects would expect to fail in many settings, so independence is best read as a strong working assumption rather than a default. The criterion that a change measure *should* be uncorrelated with the pretest (O'Connor, 1972) is itself normative and debatable: one may hold that genuine growth can legitimately depend on prior knowledge. In our data, the ngain–pretest correlation is not statistically distinguishable from zero, which is consistent with the hypothesis; we treat this as a necessary condition, noting that a null linear correlation does not by itself rule out nonlinear dependence.

Conceptually, the learning rate reflects a learner's capacity to acquire new knowledge under given instructional conditions. These conditions could be represented in a richer model that treats the learning rate as an outcome predicted by variables such as study skills, motivation, cognitive abilities, and experimental condition. Some of these predictors may themselves be related to initial knowledge, and two structurally distinct cases must be separated. In the first, a common cause *C* acts as a confounder, inducing a *spurious* pretest–ngain association through the paths *C* → pretest and *C* → ngain. Here the appropriate response is to adjust for *C*: if the association vanishes once *C* is included, the correlation was non-causal and the LR-Model hypothesis is preserved (see for example Navarrete-Ulloa, 2025; Navarrete-Ulloa and Muñoz-Rubke, 2022). In the second, a mediator *M* transmits a genuine effect of initial knowledge on the learning rate, pretest → *M* → ngain. This case is qualitatively different: the dependence is real (see for example Navarrete and Sandoval-Díaz, 2020), so the hypothesis is substantively violated, and adjusting for *M* would be inappropriate, since conditioning on a mediator blocks the very effect at issue and biases the estimated total effect. Distinguishing the two therefore requires prior substantive reasoning about the causal role of each candidate variable, not a mechanical search for controls that make the correlation disappear.

This asymmetry has a practical consequence. A residual pretest–ngain correlation may reflect either an unmodeled confounder or a genuine mediated dependence, and a researcher lacking the relevant variables cannot, from the correlation alone, distinguish “key control variables are missing” from “the LR-Model is inadequate for these data.” Conversely, when the ngain–pretest correlation is null under a pre-specified and causally justified adjustment set of variables, the evidence is consistent with the independence assumption and supports applying the LR-Model to the learning data at hand.

### 4.3 Practical implications

Neither $\bar{ng}$ nor $\hat{ng}$ should be used to estimate learning rates at the individual level (*ng*_*i*_); both are designed for group-level analysis, echoing long-standing cautions both about the unreliability of individual change scores (Cronbach and Furby, 1970) and about the sensitivity of conclusions to the choice of change metric (Bezruczko et al., 2016). The choice between them depends on the researcher's analytic goals and on the quality of the measurement instruments. It is equally important to stress that the interpretation of either estimator is highly dependent on the study design. A single-group pretest–posttest design, for instance, cannot attribute the observed change to the intervention alone: only a design with appropriate control conditions can rule out the confounders that influence learning without being manipulated. When the difference between $\bar{ng}$ and $\hat{ng}$ is large—indicating substantial measurement-error bias in $\bar{ng}$—we recommend $\hat{ng}$ as the primary estimator of the group's learning rate. The estimator $\hat{ng}$ is asymptotically unbiased and robust to measurement error, but it cannot incorporate individual-level covariates, limiting its use in analyses that require modeling individual differences (e.g., ANCOVA or linear mixed models).

This recommendation is conditional on the LR-Model's core assumption that the true learning rate is independent of initial knowledge (*X*⊥*F*). The unbiasedness of $\hat{ng}$ for μ_*F*_ rests on the identity μ_*F*_ = (μ_*Y*_−μ_*X*_)/(1−μ_*X*_) in Equation 7, which holds only under this independence. When prior knowledge and learning capacity are genuinely dependent—for instance through the mediated pathway pretest → *M* → ngain discussed in Section 4.2—this identity fails and $\hat{ng}$ no longer targets μ_*F*_: it converges instead to μ_*F*_−Cov(*X, F*)/(1−μ_*X*_). Because the validity of the *X*⊥*F* assumption is precisely what the pretest–ngain correlation debate contests, $\hat{ng}$ should be preferred as an unbiased estimator of the group learning rate only once the diagnostic steps below support the independence assumption at an adequate level of reliability.

The estimator $\bar{ng}$, while more flexible, is biased and tends to underestimate the true learning rate. Crucially, this bias is not a constant offset: by Equation 18 it is proportional to Cov(F^*^, X^*^), that is., it is systematically related to the pretest. Consequently, using individual ngains as an outcome or covariate in models that also contain pretest-correlated predictors can bias those predictors' coefficients, not merely the intercept. For this reason, $\bar{ng}$ should be used without correction only when the pretest and posttest measurements are highly reliable.

#### 4.3.1 An estimable bias correction

The results of Section 3.2 already permit a direct correction, since every term in the bias of Equation 18 is observable. Writing $ng_{i}=\left(Y_{i}^{*}-X_{i}^{*}\right)/\left(1-X_{i}^{*}\right)$ for the individual noisy ngains, $\bar{ng}=\frac{1}{n}\underset{i}{\sum }ng_{i}$, and $\bar{X^{*}}=\frac{1}{n}\underset{i}{\sum }X_{i}^{*}$, a consistent, bias-corrected group estimator is

$$\begin{array}{l} {\bar{ng}}_{\text{c}}=\bar{ng}-\frac{\hat{\text{Cov}}\left(ng_{i},X_{i}^{*}\right)}{1-\bar{X^{*}}}, \end{array}$$ (26)

where $\hat{\text{Cov}}$ denotes the sample covariance. Because the measurement errors have zero mean, $\text{𝔼}\left(X^{*}\right)=\mu_{X}$, so $1-\bar{X^{*}}$ consistently estimates *E*(1−*X*); by the continuous mapping theorem, ${\bar{ng}}_{\text{c}}\overset{p}{\to }\mu_{F}$ under the same regularity conditions used for $\hat{ng}$ finite second moments of (X^*^, Y^*^) and *E*(X^*^) < 1. Equation 26 thus recovers an approximately unbiased group learning rate while preserving the individual-level ngains needed for covariate modeling. In large, reliable samples ${\bar{ng}}_{\text{c}}$ and $\hat{ng}$ target the same quantity μ_*F*_ and should agree; a persistent gap signals residual model misspecification.

#### 4.3.2 Diagnostic procedure

We propose the following steps. First, compute $\bar{ng}$ and $\hat{ng}$ and assess their difference: a large discrepancy is a warning sign of substantial measurement-error bias. Second, report the reliability of the pretest and posttest instruments alongside both estimates, so that readers can judge how much of any observed pretest–ngain correlation is likely artifactual. Third, test the pretest–ngain correlation explicitly: a statistically negligible correlation, given adequate reliability, indicates that the data fit the LR-Model well; a significant correlation warrants investigation—either by including controls for explanatory variables (e.g., cognitive abilities, socioeconomic status) that may be confounding the pretest–ngain relationship (Navarrete-Ulloa, 2025), or by reconsidering whether the ngain transformation is appropriate for the data at hand. When bias is present but the instruments cannot be improved, the correction in Equation 26 offers an immediate partial remedy; in the Future Directions section we outline more general methods to remove the bias of $\bar{ng}$.

### 4.4 Broader methodological relevance

Beyond the specific context of ngains, this study illustrates a general analytical problem that arises whenever researchers compute nonlinear derived scores from fallible test instruments. A precedent for this problem exists in the linear case: Miyazaki et al. (2022) derived the asymptotic bias of ANCOVA treatment effect estimates caused by measurement error in the pretest, showing that even a standard linear model can produce severely distorted estimates—including sign reversals — when pretest reliability is insufficient. The present study complements that result by showing that nonlinear derived scores, such as ngains, are subject to an analogous but structurally distinct bias that is not correctable by standard ANCOVA adjustments. The bias result for $\bar{F^{*}}$ was obtained by deriving the expected value of a ratio under additive measurement noise, using Jensen's inequality to establish the sign of the bias. This approach is not specific to the ngain ratio: the same framework could be applied to other commonly used derived scores in educational and psychological measurement—such as effect size indices, gain ratios, or proportion-correct transformations—wherever a nonlinear function of observed scores is used to estimate a population quantity. The present paper thus offers a methodological template that may be useful beyond the ngains debate, and we encourage future work to explore analogous bias structures in related metrics.

### 4.5 Limitations

This study has three key limitations that qualify its conclusions. First, the assumption of additive Gaussian measurement errors can produce scores outside the valid range [0, 1]. In our simulations, discarded data amount to fewer than 0.1%, but we are aware that this might be a problem when the mean of *X* or *F* is close to the boundaries. In such cases, the simulated bias estimates may understate the true bias, since out-of-range values are discarded. This boundary constraint limits the generalizability of the simulation results to settings where pretest and posttest means are near ceiling or floor. More fundamentally, the sign-of-bias result is stated for admissible (bounded) measurement errors: under exact Gaussian errors the mean *E*[(1−^X^*^)−1^]—and hence *E*(F^*^)—does not exist, so our finite-sample bias statements should be read as applying to the truncated error model that the discarding rule in Table 1 operationalizes.

Second, an important nuance emerges when the ngain transformation is applied to data in which prior knowledge and learning capacity are not, in fact, independent. Our framework establishes that measurement error induces a negative component in the pretest-ngain correlation, arising purely from the shared pretest error in X^*^ and F^*^. In real educational settings, however, a positive component may coexist with it: when latent variables such as cognitive ability, socioeconomic status, or prior instructional exposure jointly influence both initial knowledge and the learning ability, they might induce a genuine positive dependence between true pretest scores and true learning rates. The observed correlation *corr*(F^*^, X^*^) is then the net result of two opposing mechanisms—a spurious negative term from measurement error and a substantive positive term from confounding—which may partially or wholly cancel. This has a consequence that is easy to overlook: a researcher who observes a positive, null, or attenuated pretest-ngain correlation cannot conclude that measurement error is absent, nor that the ngain transformation has successfully controlled for prior knowledge. A positive observed correlation may reflect confounding strong enough to overwhelm the error-induced negative term, while a near-zero correlation may mask the coincidental cancellation of two effects of opposite sign. Consequently, the sign and magnitude of the observed correlation are informative about the ngain transformation only once both measurement reliability and potential confounders have been explicitly accounted for.

Third, and more fundamentally, the entire framework is grounded in Classical Test Theory (CTT), which models observed scores as true scores plus additive error. CTT has well-known limitations: it does not model the probability of a correct item response as a function of latent ability, it assumes equal error variances across score levels, and it is less suited than Item Response Theory (IRT) for analyzing tests with items that differ in difficulty or discrimination. Whether the bias result for $\bar{F^{*}}$ holds—or changes in sign or magnitude—under IRT-based scoring is an open question (Bezruczko et al., 2016) that the present framework cannot answer. Future work should extend these results to IRT models, which would provide more accurate characterizations of measurement error at the score boundaries and better-calibrated reliability estimates.

### 4.6 Future directions

Our results open several concrete avenues for future research. We have a trade-off between two estimators. On the one hand, $\hat{F^{*}}$ is assymptotically unbiased to estimate group level change, but it has no way to add individual variables into the analysis. On the other hand, $\bar{F^{*}}$ is negatively biased but has the ability to add individual variables into the analysis. When $\bar{F^{*}}$ is used as an outcome in a model such as ANCOVA, this bias propagates into the estimated treatment effect and covariate coefficients—and, because the observed pretest enters the model as a fallible covariate, including it does not fully absorb the bias (Miyazaki et al., 2022). As future research, it is desirable that the two estimation methods studied here can be combined into one method with the ability to input individual information into the analysis and proves unbiased estimates. For example, as future research, we propose develop and compare a family of correction strategies that combine complementary tools: (i) a closed-form analytical bias expression that anchors the correction Cov(F^*^, X^*^)/*E*(1−*X*) at the observed error level; (ii) the asymptotically unbiased estimator $\hat{F^{*}}$, used as a group-level constraint or extrapolation anchor; and (iii) an extrapolation method which corrects all model parameters simultaneously when bias propagates heterogeneously across covariate levels. For the case of (iii), we propose using the SIMEX method (Cook and Stefanski, 1994) which is a simulation-based method of inference for parametric measurement error models in which the measurement error variance is known or at least well estimated. Developing, comparing, and validating these correction strategies in simulated and realistic educational datasets is a natural and important next step. Beyond bias correction, future research should also examine how the bias-flexibility trade-off identified here interacts with common analytic choices in education research, such as the use of multilevel models, propensity score matching, or latent growth curve analysis, all of which rely on ngain-like derived scores as outcome variables.

## 5 Conclusion

This study provides the first rigorous statistical analysis of the two most commonly used methods for estimating group-level normalized gains: the average ngain $\bar{ng}$ and the ngain of the average learner $\hat{ng}$. Four main conclusions emerge from the theoretical and simulation results.

First, in the absence of measurement errors, both estimators are unbiased, and any observed discrepancy between them is due solely to sampling variation. Second, in the presence of measurement errors, a fundamental asymmetry arises: $\hat{ng}$ remains asymptotically unbiased while $\bar{ng}$ acquires a systematic negative bias that is directly proportional to the covariance between observed pretest scores and observed ngains—a covariance that is itself induced by measurement error. Third, this spurious covariance provides a statistical explanation for the persistent pretest-ngain correlations documented in the literature: they need not reflect a genuine dependence between prior knowledge and learning capacity, but may likely be reflecting insufficient measurement reliability. Fourth, Bao's (Bao, 2006) characterization of the learning process based on the sign of $\left(\bar{ng}-\hat{ng}\right)$ is reinterpreted: what Bao attributed to differential learning across pretest levels is more parsimoniously explained by the bias of $\bar{ng}$ under measurement error.

Together, these findings offer a coherent account of two decades of conflicting results in the ngains literature, establish a principled basis for choosing between the two estimators, and identify a concrete path toward bias-corrected estimation methods. Further research is needed to extend this framework to Item Response Theory models, to develop and validate practical bias corrections, and to examine how the bias-flexibility trade-off identified here affects broader analytic strategies in educational research.

## Data Availability

The datasets presented in this study can be generated through the code in online repositories. The names of the repository and access number can be found below: https://doi.org/10.17605/OSF.IO/AMF3D.
